# CSF tau368/total-tau ratio reflects cognitive performance and neocortical tau better compared to p-tau181 and p-tau217 in cognitively impaired individuals

**DOI:** 10.1186/s13195-022-01142-0

**Published:** 2022-12-22

**Authors:** Joel Simrén, Wagner S. Brum, Nicholas J. Ashton, Andrea L. Benedet, Thomas K. Karikari, Hlin Kvartsberg, Emma Sjons, Firoza Z. Lussier, Mira Chamoun, Jenna Stevenson, Robert Hopewell, Vanessa Pallen, Keqiang Ye, Tharick A. Pascoal, Henrik Zetterberg, Pedro Rosa-Neto, Kaj Blennow

**Affiliations:** 1 Department of Psychiatry and Neurochemistry, Institute of Neuroscience and Physiology, Sahlgrenska Academy, University of Gothenburg, Gothenburg, Sweden; 2 Clinical Neurochemistry Laboratory, Sahlgrenska University Hospital, Gothenburg, Sweden; 3 Graduate Program in Biological Sciences: Biochemistry, Universidade Federal do Rio Grande do Sul (UFRGS), Porto Alegre, Brazil; 4 Wallenberg Centre for Molecular and Translational Medicine, University of Gothenburg, Gothenburg, Sweden; 5 Maurice Wohl Clinical Neuroscience Institute, Institute of Psychiatry, Psychology and Neuroscience, King’s College, London, London, UK; 6 Translational Neuroimaging Laboratory, McGill University Research Centre for Studies in Aging, Alzheimer’s Disease Research Unit, Douglas Research Institute, Le Centre intégré universitaire de santé et de services sociaux (CIUSSS) de l’Ouest-de-l’Île-de-Montréal; Department of Neurology and Neurosurgery, McGill University, Montreal, Canada; 7 Department of Psychiatry, University of Pittsburgh, Pittsburgh, PA USA; 8 Montreal Neurological Institute, Montreal, QC Canada; 9 Department of Pathology and Laboratory Medicine, Emory University School of Medicine, Atlanta, GA USA; 10 Department of Neurology and Psychiatry, University of Pittsburgh, Pittsburgh, USA; 11 Department of Neurodegenerative Disease, Institute of Neurology, University College London, London, UK; 12 UK Dementia Research Institute, University College London, London, UK; 13 Hong Kong Center for Neurodegenerative Diseases, Hong Kong, China

## Abstract

**Introduction:**

Cerebrospinal fluid (CSF) tau biomarkers are reliable diagnostic markers for Alzheimer’s disease (AD). However, their strong association with amyloid pathology may limit their reliability as specific markers of tau neurofibrillary tangles. A recent study showed evidence that a ratio of CSF C-terminally truncated tau (tau368, a tangle-enriched tau species), especially in ratio with total tau (t-tau), correlates strongly with tau PET tracer uptake. In this study, we set to evaluate the performance of the tau368/t-tau ratio in capturing tangle pathology, as indexed by a high-affinity tau PET tracer, as well as its association with severity of clinical symptoms.

**Methods:**

In total, 125 participants were evaluated cross-sectionally from the Translational Biomarkers of Aging and Dementia (TRIAD) cohort (21 young, 60 cognitively unimpaired [CU] elderly [15 Aβ+], 10 Aβ+ with mild cognitive impairment [MCI], 14 AD dementia patients, and 20 Aβ− individuals with non-AD cognitive disorders). All participants underwent amyloid and tau PET scanning, with [^18^F]-AZD4694 and [^18^F]-MK6240, respectively, and had CSF measurements of p-tau181, p-tau217, and t-tau. CSF concentrations of tau368 were quantified in all individuals with an *in-house* single molecule array assay.

**Results:**

CSF tau368 concentration was not significantly different across the diagnostic groups, although a modest increase was observed in all groups as compared with healthy young individuals (all *P* < 0.01). In contrast, the CSF tau368/t-tau ratio was the lowest in AD dementia, being significantly lower than in CU individuals (Aβ−, *P* < 0.001; Aβ+, *P* < 0.01), as well as compared to those with non-AD cognitive disorders (*P* < 0.001). Notably, in individuals with symptomatic AD, tau368/t-tau correlated more strongly with [^18^F]-MK6240 PET SUVR as compared to the other CSF tau biomarkers, with increasing associations being seen in brain regions associated with more advanced disease (isocortical regions > limbic regions > transentorhinal regions). Importantly, linear regression models indicated that these associations were not confounded by Aβ PET SUVr. CSF tau368/t-tau also tended to continue to become more abnormal with higher tau burden, whereas the other biomarkers plateaued after the limbic stage. Finally, the tau368/t-tau ratio correlated more strongly with cognitive performance in individuals with symptomatic AD as compared to t-tau, p-tau217 and p-tau181.

**Conclusion:**

The tau368/t-tau ratio captures novel aspects of AD pathophysiology and disease severity in comparison to established CSF tau biomarkers, as it is more closely related to tau PET SUVR and cognitive performance in the symptomatic phase of the disease.

**Supplementary Information:**

The online version contains supplementary material available at 10.1186/s13195-022-01142-0.

## Introduction

The aggregation and propagation of hyperphosphorylated tau is a key feature of Alzheimer’s disease (AD) pathogenesis, and the deposition of these species into neurofibrillary tangles is one of the defining hallmarks observed at neuropathological examination of AD [1]. Altered tau phosphorylation and secretion can be detected in cerebrospinal fluid (CSF), through measurement of tau phosphorylated at certain amino acid residues, with threonine 181 (p-tau181) being most commonly used, and total mid-region-containing species of tau (t-tau) [2]. Recent research suggest that tau phosphorylated at other sites, such as threonine 217 (p-tau217), are comparable [3, 4] and, in some studies, more accurate in detecting these alterations [5]. Biomarker modeling studies suggest that the abnormal release of these species occurs early in the disease process—before neurodegeneration and symptomatic disease [6]. Instead, the alterations in soluble tau co-occur with early accumulation of amyloid-β (Aβ) [3] and before tau positron emission tomography (PET) becomes abnormal [7, 8]. It is only rather late in the disease process, close to clinical disease onset, that significant tau aggregation can be seen by tau PET outside medial temporal brain regions [9]. Taken together, this may at least partly explain the fact that current fluid biomarkers of tau pathology present relatively strong associations with amyloid PET but more modest associations with tau PET. Furthermore, changes in the proteolytic processing of tau is likely important for its tendency to aggregate [10]. In line with this, tau exists as fragments [11–13], rather than as an intact protein, both in tangles and in the CSF. The importance of tau proteolysis for the propensity of the peptides to become hyperphosphorylated and subsequently aggregate was demonstrated in a study where asparagine endopeptidase (AEP, also called legumain) was shown to cleave tau at amino acid 368 (N368) in an age-dependent manner [14]. This fragment—which lacks the C-terminal tail of tau—resulted in tau aggregation and phosphorylation as well as neurodegeneration and was present in neurofibrillary tangles in AD brains [14]. To test the hypothesis that C-terminal tau in CSF reflects tangle pathology, we developed an assay targeting tau368. We showed that it decreased across the AD continuum and correlates with tau PET signal, especially when used in a ratio with t-tau [15]. Thus, in this study, we aimed to characterize how tau368/t-tau reflects pathological neurofibrillary tau deposition as determined by PET in a larger cohort, to investigate how it relates to cognitive function, and how it can be used in differential diagnostics. Furthermore, we explored if these features are different when comparing to fluid biomarkers that are currently used to index tau pathology (t-tau, p-tau217 and p-tau181).

## Methods

### Participants

We included individuals from the Translational Biomarkers of Aging and Dementia (TRIAD) cohort, McGill University, Canada. In the TRIAD cohort, all participants had CSF and PET (amyloid and tau) biomarkers and detailed clinical and cognitive assessments, including Mini-Mental State Examination (MMSE), Montreal Cognitive Assessment (MoCA), and the clinical dementia rating (CDR) tests. The TRIAD cohort consisted of cognitively unimpaired (CU) young (median age, years [IQR]) (23.1 [22.7–24.1]) and CU elderly (72.5 [67.7–76.7]) as well as mild cognitive impairment (MCI), AD, and non-AD dementia patients. CU participants had an MMSE score > 24 and a CDR score of 0. MCI participants had a CDR score of 0.5, subjective and objective impairments in cognition, but preserved activities of daily living. AD dementia patients had a CDR score ≥ 0.5 and met the National Institute on Aging and the Alzheimer’s Association criteria for probable Alzheimer’s disease determined by a physician [16] and were Aβ PET positive. The non-AD participants had CDR score ≥ 0.5, were Aβ PET-negative, and had clinical diagnosis of FTD (*n* = 7), progressive supranuclear palsy (*n* = 1), MCI (*n* = 12), or clinically diagnosed AD (*n* = 2). In the TRIAD cohort, participants were excluded if they had active substance abuse or inadequately treated conditions, recent head trauma or major surgery, or if they presented safety contraindication for the study procedures.

### Biochemical analysis

The commercial fully automated LUMIPULSE G1200 (Fujirebio) was used to measure CSF p-tau181, t-tau, as previously described [17]. CSF p-tau217 was measured using an *in-house* single molecule array (Simoa) assay, as detailed in Karikari et. al. [18] Tau368 was measured using a validated *in-house* Simoa assay, which has been detailed elsewhere [15]. Briefly, an anti-tau368 antibody was used as capture antibody, whereas K9JA (a rabbit polyclonal antibody against 243-441, Sigma) was used as detector. All biochemical analyses were performed at the Clinical Neurochemistry Laboratory at the Sahlgrenska University hospital, Mölndal, Sweden. Of 125 individuals, CSF p-tau217 was available for 116 of the participants, while the other biomarkers were available for all individuals.

### Imaging methods

All individuals in the TRIAD cohort were assessed with Siemens 3T MRI as well as Aβ [^18^F]-AZD4694 PET and tau [^18^F]-MK-6240 PET acquired with a Siemens High Resolution Research Tomograph. [^18^F]-MK-6240 images were acquired at 90–110 min after the intravenous bolus injection of the radiotracer [19, 20]. Aβ [^18^F]-AZD4694 PET images were acquired at 40–70 min after the intravenous bolus injection of the radiotracer [19, 20]. The PET images were spatially smoothed to achieve a final 8-mm full width at half maximum resolution and were processed using a previously described pipeline [19, 20]. [^18^F]-MK6240 images were stripped off the meninges before smoothing, as described elsewhere [20]. [^18^F]-MK6240 standard uptake value ratio (SUVR) was measured regionally based on the anatomical brain regions proposed by Braak and Braak as follows: Braak I (transentorhinal), Braak II (entorhinal and hippocampus), Braak III (amygdala, parahippocampal gyrus, fusiform gyrus, lingual gyrus), Braak IV (insula, inferior temporal, lateral temporal, posterior cingulate, and inferior parietal), Braak V (orbitofrontal, superior temporal, inferior frontal, cuneus, anterior cingulate, supramarginal gyrus, lateral occipital, precuneus, superior parietal, superior frontal, rostro medial frontal), and Braak VI (paracentral, postcentral, precentral, and pericalcarine). In this study, the six Braak stages were collapsed to three main stages (as proposed by Braak and Braak) [21]: transentorhinal, limbic, and isocortical regions, approximating Braak stages I–II, III–IV, and V–VI of neurofibrillary tangle (NFT) pathology, respectively. The Desikan-Killiany-Tourville atlas [22] was used to define the regions-of-interest for the PET Braak-like stages. Tau positivity was defined as 2.5 standard deviations (SD) higher than the mean SUVR of cognitively unimpaired young individuals for each respective region of interest. Determination of individual Braak staging was performed using an automatic pipeline in a hierarchical fashion, where later stages can only be achieved if the individual is positive for the previous stages; otherwise, the participant was considered Braak stages discordant. Discordant individuals were not included in the analyses performed in this paper (*n* = 12) presented in Fig. 3, whereas they were included in all other analyses. Individuals negative for tau PET uptake in all aforementioned regions-of-interest were classified as in vivo Braak stage 0. Further descriptions of the in vivo Braak-like staging can be found elsewhere [20]. Global [^18^F]-AZD4694 SUVR was derived from averaging retention in the precuneus, the cingulate, inferior parietal, medial prefrontal, lateral temporal, and orbitofrontal cortices. Aβ [^18^F]-AZD4694 SUVR positivity was based on visual assessment [23]. Structural MRI data were acquired using a Siemens 3T scanner using a standard head coil. Hippocampal volume was assessed using FreeSurfer version 6.0 using the Desikian–Killiany–Touriner atlas gray matter segmentation. Hippocampal volume was adjusted for intracranial volume.

### Statistical analysis

Normality was tested by determining kurtosis and skewness, as well as with the Shapiro-Wilks test. All fluid biomarkers except for tau368/t-tau were non-normally distributed. Thus, non-parametric methods were selected. In analyses of only AD dementia and prodromal AD, tau368/t-tau, tau, and amyloid PET SUVR measures were roughly normally distributed (skewness and kurtosis ± 1), but the other fluid biomarker measures were log-transformed_10_ due to non-normality in linear regression models. Group-wise comparisons of continuous variables were performed using Kruskal-Wallis test or Mann-Whitney *U* test where appropriate and were corrected for multiple comparisons using false discovery rate (FDR). Fisher’s exact test was used to compare categorical variables across groups. Correlations between biomarkers and other continuous variables were tested with Spearman rank correlation. Linear regression models were used to evaluate whether amyloid influenced the association between tau PET and tau368/t-tau. These models had CSF tau368/t-tau as the response variable and had tau PET alone or tau PET and amyloid PET as predictors. Akaike information criteria with a correction for small sample sizes (AICc; used when n divided by number of parameters are less than about 40) was used to assess the model fit, with a penalty for a more complex model [24]. Areas under the curve (AUC) was assessed using receiver operating characteristics (ROC) analysis. Differences in AUCs were evaluated with bootstrapping (*n* = 2000), using the pROC package in R [25]. In the comparisons including p-tau217, only individuals with CSF p-tau217 measured were included. *Z*-scores of the fluid biomarkers were calculated using the CU elderly individuals in Braak stage 0 as a reference. Statistical analyses were performed using GraphPad Prism v.9.2.0 (La Jolla, CA, USA) and R Statistical Software (https://www.r-project.org/).

## Results

### Participant characteristics

We studied 125 (72 [58%] women) individuals (healthy young individuals [*n* = 21], CU Aβ− [*n* = 45] CU Aβ+ [*n* = 15], MCI Aβ+ [*n* = 10; henceforth used interchangeably with prodromal AD], AD dementia [*n* = 14], non-AD cognitive impairment [*n* = 20]). Participant’s characteristics are summarized in Table 1. There was a significant association between age and t-tau (Spearman rho (*ρ*) = 0.37, *P* < 0.001), p-tau217 (*ρ* = 0.30, *P* = 0.001), p-tau181 (*ρ* = 0.39, *P* < 0.001), and tau368 (*ρ* = 0.45, *P* < 0.001), but not tau368/t-tau. After excluding the young individuals, only tau368 remained significantly associated with age (*ρ* = 0.23, *P* < 0.05). There were no significant sex-differences in tau368 or in tau368/t-tau. Tau368/t-tau was lower in *APOE* ε4 carriers as compared to non-carriers (*P* < 0.05). In addition, tau368 correlated both with both t-tau, p-tau217, and p-tau181 in all individuals, as well as when stratified into participants with symptomatic AD vs all others (all *ρ* = 0.6–0.9, *P* < 0.001).

**Table 1:** Participants characteristics

	Young (*n* = 21)	CU Aβ− (*n* = 45)	CU Aβ+ (*n* = 15)	MCI Aβ+ (*n* = 10)	AD (*n* = 14)	Non-AD (*n* = 20)	*P*-value
Age, years	23.1 (22.7–24.1)	71.7 (67.6–75.3)	74.0 (69.2–76.9)	73.6 (70.1–78.0)	67.0 (62.3–69.3)	69.5 (62.5–73.5)	.109^Ɨ^
Sex, female/male (% females)	12/9 (57)	26/19 (58)	11/4 (73)	5/5 (50)	7/7 (50)	11/9 (55)	.832
Education, years	17.0 (16.0–18.0)	15.0 (12.0–17.0)	14.0 (12.0–15.0)	17.0 (13.8–18.0)	16.0 (14.3–16.0)	12.0 (10.8–17.0)	< .001
*APOE ε4* status, pos./neg. (% pos.)	3/18 (14)	14/31 (31)	3/12 (20)	6/4 (60)	9/5 (64)	5/15 (25)	< .001
MoCA score^a^	28.5 (28.0–29.0)	28.0 (27.0–29.0)	28.0 (27.0–29.0)	25.5 (24.3–26.0)	12.5 (8.25–17.5)	25.0 (22.0–27.0)	< .001
Aβ PET SUVR	1.18 (1.12–1.20)	1.25 (1.19–1.34)	2.00 (1.67–2.28)	2.39 (2.19–2.69)	2.49 (2.23–2.75)	1.28 (1.20–1.40)	< .001
Pos./neg. Braak 1–4 (% pos)	0/21 (0)	4/41 (9)	7/8 (47)	9/1 (90)	14/0 (100)	3/17 (15)	< .001
CSF P-tau181 (pg/mL)	21.5 (18.2–27.0)	34.2 (28.0–43.1)	60.3 (43.5–68.8)	100 (91.1–111)	77.4 (62.3–116)	35.7 (24.0–42.7)	< .001
CSF P-tau217 (pg/mL)^b^	2.43 (1.94–4.01)	4.21 (3.44–6.14)	14.5 (9.45–18.2)	25.65 (23.7–30.2)	21.5 (17.0–43.1)	5.23 (3.73–6.83)	< .001
T-tau (pg/mL)	203 (158–219)	304 (258–364)	363 (293–458)	635 (533–672)	517 (413–788)	283 (226–402)	< .001
Tau368 (pg/mL)	24.3 (22.2–29.1)	35.8 (30.1–43.1)	42.2 (34.6–46.0)	46.1 (40.1–62.5)	36.0 (29.8–48.8)	34.9 (27.9–39.9)	.126
Tau368/t-tau	0.135 (0.112–0.142)	0.121 (0.112–0.142)	0.115 (0.0935–0.120)	0.0804 (0.0727–0.0918)	0.0645 (0.0475–0.0736)	0.122 (0.100–0.136)	< .001

Data shown as median (IQR; interquartile range) or *n* (%), as appropriate. Continuous variables were compared using Kruskal-Wallis test of Fischer’s exact test to compare frequencies of categorical variables between groups. *Abbreviations*: *AD* Alzheimer’s disease, *CU* cognitively unimpaired, *MCI* mild cognitive impairment, *P-tau181/217* phosphorylated tau 181/217, *T-tau* total tau, *CSF* cerebrospinal fluid, *SUVR* standardized uptake value ratio, *MoCA* Montreal cognitive assessment

^a^MoCA scores missing for seven young participants, four CU individuals and three non-AD individuals

^b^P-tau217 missing for four young individuals, two CU (one CU+), one AD and two non-AD

^Ɨ^When excluding young individuals

### Comparison between diagnostic groups

**Fig. 1:**
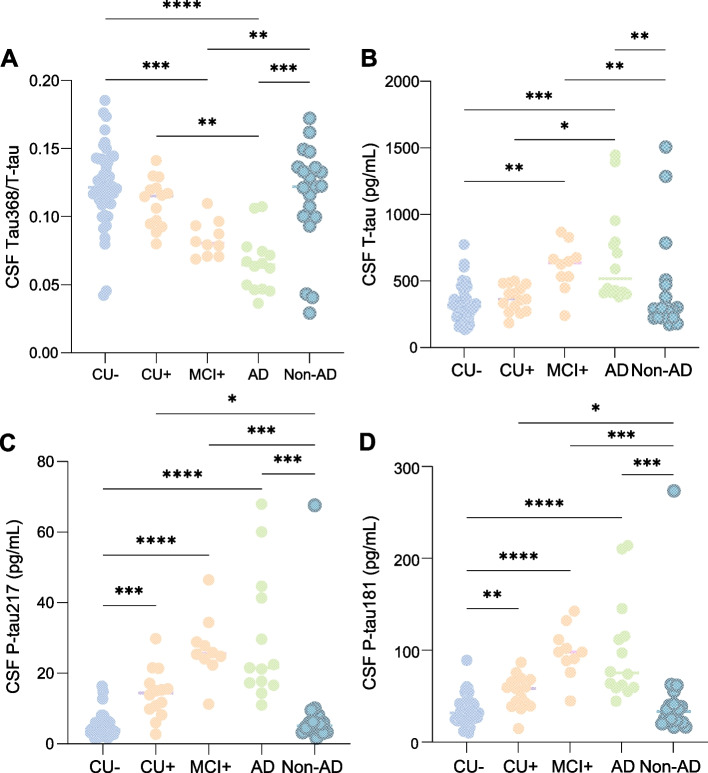
Tau368/t-tau is decreased in symptomatic AD. Baseline comparisons of CSF concentrations of p-tau181, p-tau217, tau368/T-tau, and T-tau in all diagnostic groups. Young individuals are not included in graphs for illustrative purposes but were included in statistical analyses. AD, Alzheimer’s disease; CU, cognitively unimpaired; MCI, mild cognitive impairment; P-tau181/217, phosphorylated tau 181/217; T-tau, total tau; CSF, cerebrospinal fluid. All *P*-values are derived from the Kruskal-Wallis test, adjusted for false discovery rate (FDR) using the Benjamini-Hochberg method. Tau368/t-tau, p-tau181, T-tau (*n* = 125), CSF p-tau217 (*n* = 116). *P < 0.05; ** *P* < 0.01; *** *P* < 0.001; **** *P* < 0.0001

For tau368, higher concentrations were seen in all groups as compared to young healthy individuals, but no other significant group-differences were seen (Supplementary Figure 1). However, when using tau368 in a ratio with t-tau, individuals with AD dementia had a significantly lower ratio than CU elderly individuals, with (*P* < 0.01) or without (*P* < 0.001) amyloid pathology, as well as compared to those with non-AD cognitive disorders (*P* < 0.001; Fig 1A). A decrease could be observed also in prodromal AD, when comparing with CU- elderly (*P* < 0.01) and non-AD cognitive disorders (*P* < 0.01). Tau368/t-tau separated AD dementia vs non-AD with an area under the curve (AUC) of 0.84 (95% CI 0.68–0.99) and prodromal AD vs. non-AD with and AUC of 0.82 (95% CI 0.66–0.98). The respective accuracies were numerically higher than t-tau alone (AD dementia vs. non-AD 0.82, 95% CI 0.67–0.97; prodromal AD vs non-AD: 0.80, 95% CI 0.63–0.97) but numerically lower than p-tau181 (AD dementia vs. non-AD 0.92, 95% CI 0.8–1.0; prodromal AD vs non-AD: 0.93, 95% CI 0.83–1.0) and p-tau217 (AD dementia vs. non-AD 0.95, 95% CI 0.85–1.0 and prodromal AD vs non-AD: 0.94, 95% CI 0.84–1.0). No statistical differences between the respective AUCs were seen (all *P* >0.05).

### Associations with tau PET

**Fig. 2:**
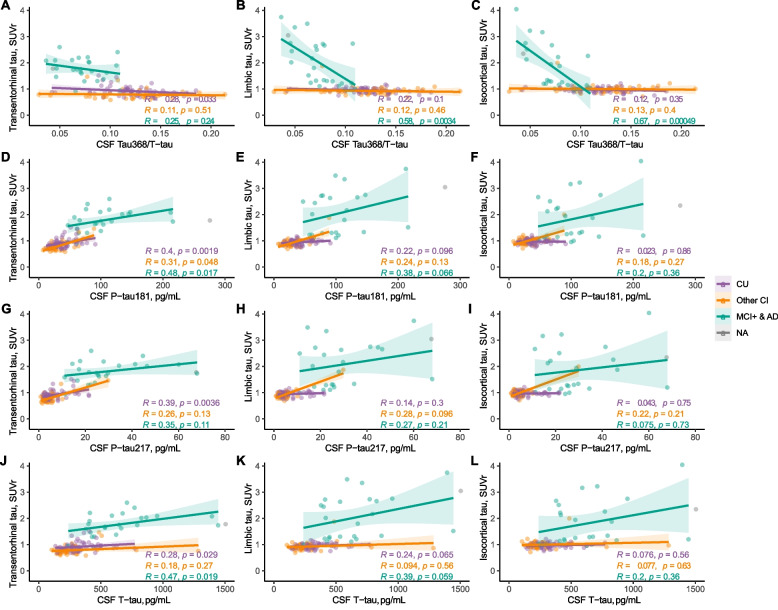
Tau368/t-tau is associated with neocortical tau load in symptomatic AD patients. Associations between ^18^F-MK-6240 PET SUVR in a priori-defined in vivo Braak stages (I–II, III–IV, and V–VI) and CSF **A**–**C** tau368/t-tau, **D**–**F** p-tau217, and **G**–**I** p-tau181 and **J**–**L** t-tau. AD, Alzheimer’s disease; CU, cognitively unimpaired; MCI, mild cognitive impairment; CI, cognitive impairment; P-tau181/217, phosphorylated tau 181/217; T-tau, total tau; CSF, cerebrospinal fluid; SUVR, standardized uptake value ratio. All *P*-values and R coefficients are derived from Spearman correlations. One clear outlier, shown in grey and labeled NA, was excluded from the statistical analysis but remain in the graph. Tau368/t-tau, p-tau181, T-tau (*n* = 125), CSF p-tau217 (*n* = 116)

As it has been hypothesized that tau368/t-tau well reflects tau accumulation, we evaluated its relationship with tau PET and then compared it with the associations between tau PET and t-tau, p-tau181, and p-tau217. When investigating the continuous relationship between the SUVR of [^18^F]-MK-6240 PET in in vivo Braak-like regions and fluid biomarkers, as seen in previous studies [4], all biomarkers correlated moderately to strongly with tau PET uptake in the whole group. The strongest associations were seen for CSF p-tau181 and p-tau217 in all in vivo Braak-like regions (Supplementary Table 1). However, when stratifying the groups into symptomatic AD, CU, and other CI, we found that tau368/t-tau was increasingly associated with tau PET SUVR in brain regions commonly being affected later by fibrillar tau deposition in participants with symptomatic AD (limbic regions: *ρ* = − 0.58, *P* < 0.01; isocortical regions: *ρ* = − 0.67, *P* < 0.001), with no strong associations observed between tau368/t-tau and tau PET SUVR in any of the brain regions in the groups encompassing other participants (Fig. 2A–C). Regarding the associations of other CSF tau biomarkers with tau PET in the symptomatic AD group, p-tau181 and t-tau only associated with SUVR in the transentorhinal (corresponding to in vivo Braak I–II) composite region (p-tau181: *ρ* = 0.48, *P* < 0.05, *t*-tau: *ρ* = 0.47, *P* < 0.05), while p-tau217 was not associated with SUVR in any of the tau PET brain regions (Fig. 2D–L). Further, tau368 when instead used in a ratio with p-tau181 presented very similar findings as compared with tau368/t-tau, whereas no significant associations were seen for tau368/p-tau217 (Supplementary Table 2). Interestingly, among these participants, there were strong correlations between lower tau368/t-tau and younger age. Similar associations were seen between limbic and isocortical tau as indexed with tau PET (Supplementary Figure 2). Further, when evaluating the associations of CSF tau biomarkers with tau PET SUVR in the groups encompassing the other participants (all CU, other CI), no strong significant associations were observed for tau368/t-tau, while several significant associations were observed for p-tau181, p-tau217, and t-tau.

When using linear models to investigate whether these associations observed in the symptomatic AD group could be driven by Aβ pathology, we found that the relationship between tau368/t-tau and tau PET signal in limbic regions was not affected when amyloid PET SUVr was included in linear regression models (Table 2; corresponding tables for p-tau181, p-tau217, and t-tau are available in Supplementary Tables 3-5).

**Table 2:** The relationship between tau368/t-tau and tau PET symptomatic AD is not affected by amyloid

Independent variables		*R*^2^	Adj. *R*^2^	AICc
Amyloid composite		7%	3%	− 183.2
Transentorhinal tau (I–II)	Tau	6%	1%	− 182.8
	Amyloid + Tau	10%	2%	− 181.1
Limbic tau (III–IV)	Tau	32%	29%	− 190.6^a^
	Amyloid + Tau	33%	27%	− 188.2
Isocortical tau (V–VI)	Tau	41%	38%	− 193.9^a^
	Amyloid + Tau	42%	36%	− 191.3

Associations between tau PET SUVr and tau368/t-tau in the hierarchical Braak stages demonstrate that the associations strengthen in the later Braak stages. *R*^2^ and adjusted *R*^2^, measures of how the outcome variable is explained by the models, are shown. Akaike information criteria with correction for small sample sizes (AICc) was calculated to determine the best fitting model, accounting for the complexity of the model. An AIC < 2 compared to another model indicates less information loss, and thus a better model^a^

*P < 0.01

**Fig. 3:**
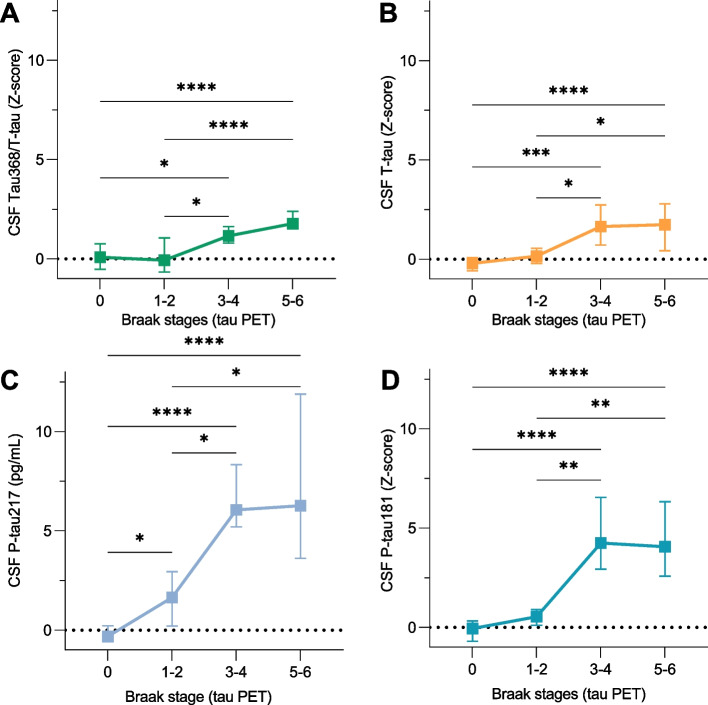
Tau368/t-tau abnormality is associated with in vivo Braak staging. Trajectories of **A** tau368/t-tau, **B** t-tau, and **C** p-tau217 and **D** p-tau181 increase with respect to in vivo Braak staging (I–II, III–IV, and V–VI) as indexed by ^18^F-MK-6240 PET. Medians are represented by shapes and error bars indicate interquartile range. *Z*-scores are derived from cognitively unimpaired elderly participants at Braak stage 0. Young individuals are not included in this graph. *N* = 56, 14, 8, and 14 individuals were classified as being at Braak stages 0, I–II, III–IV, and V–VI, respectively. Discordant cases *n* = 12 were excluded from this but included in all other analyses in this paper. All *P*-values are derived from the Kruskal-Wallis test, adjusted for false discovery rate (FDR) using the Benjamini-Hochberg method. *P < 0.05; ** *P* < 0.01; *** *P* < 0.001; *P* < 0.0001

Furthermore, we investigated the trajectories of tau368/t-tau across in vivo Braak-like brain stages, by comparing the *Z*-scored CSF tau biomarkers of individuals in that were considered tau PET positive in transentorhinal, limbic, and isocortical regions, respectively. There were pronounced increases between the transentorhinal and limbic stages especially for p-tau181 and p-tau217 (Fig. 3C, D). Although there was no statistically significant change between individuals in the limbic vs. the isocortical stages, tau368/t-tau seemingly continues to become more abnormal as the tau pathology progresses, unlike p-tau217 and 181 and t-tau, which plateau as the tau pathology becomes widespread across the neocortex (Fig. 3A–D).

Regarding the discriminative ability of tau368/t-tau for tangle pathology, it identified tau positivity in transentorhinal regions (Braak I-II) with an AUC of 0.85 (95% CI 0.77-0.91), as compared to 0.93 (95% CI 0.88–0.98), 0.94 (95% CI 0.89–0.99), and 0.87 (95% CI 0.80–0.93) for p-tau181, p-tau217, and t-tau, respectively (Supplementary Figure 3). This was significantly lower as compared to p-tau181 (*P* < 0.05; p-tau217, *P* = 0.06; t-tau, *P* = 0.48). Individuals with tau PET positivity at least in limbic regions (Braak III–IV) were identified with an accuracy of 0.92 (0.86–0.97) using tau368/t-tau, with AUCs being 0.98 (95% CI 0.95–1.0; *P* < 0.05 vs. tau368/t-tau), 0.99 (95% CI 0.97–1.0; *P* < 0.05 vs. tau368/t-tau), and 0.93 (95% CI 0.88–0.98; *P* = 0.47 vs. tau368/t-tau) for p-tau181, p-tau217, and t-tau. In the isocortical stages (Braak V-VI), however, tau368/t-tau (95% CI 0.93, 0.87–0.98) performed similarly (all *P* > 0.05 vs tau368/t-tau) as compared to p-tau181 (0.93, 95% CI 0.88–0.98), p-tau217 (0.94, 95% CI 0.90–0.99), and t-tau (0.89, 95% CI 0.83–0.95). Of note, there was no association between tau368/t-tau and hippocampal volume (Supplementary Figure 4).

### Association with cognitive measures

**Fig. 4:**
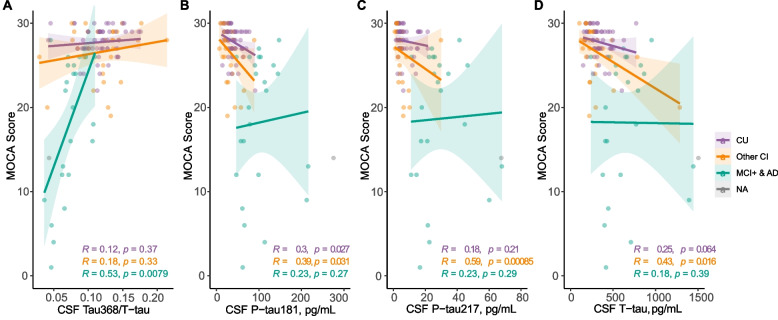
Tau368/t-tau is associated with cognitive performance in symptomatic AD patients. Associations between cognitive performance as indexed by MoCA score and CSF **A** tau368/t-tau, **B** p-tau217, and **C** p-tau181 and **D** t-tau. Participants are stratified into Aβ+ MCI and AD dementia vs. all others. AD, Alzheimer's disease; MCI, mild cognitive impairment; CI, cognitive impairment; P-tau181/217, phosphorylated tau 181/217; T-tau, total tau; CSF, cerebrospinal fluid; MoCA, Montreal cognitive assessment. All *P*-values and R coefficients are derived from Spearman correlations. One clear outlier, shown in grey and labeled NA, was excluded from the statistical analysis but remain in the graph. Tau368/t-tau, p-tau181, T-tau (*n* = 107), CSF p-tau217 (*n* = 100)

As tau368/t-tau was significantly associated with tau PET tracer uptake in brain regions associated with more advanced tau pathology, we hypothesized that it may have a closer relationship with cognitive performance than core CSF biomarkers. Using MoCA as a measure of global cognition, there were expected correlations in the whole group with all CSF biomarkers, being the strongest for p-tau181 and p-tau217 (*ρ* = − 0.52 and *ρ* = − 0.55, respectively) (Supplementary Table 6). However, the degree of cognitive impairment was more accurately reflected by tau368/t-tau (*ρ* = 0.53, *P* < 0.01) than p-tau181 (*ρ* = 0.23, *P* = 0.27), p-tau217 (*ρ* = 0.23, *P* = 0.29), t-tau (*ρ* = 0.18, *P* = 0.39), and ratios of tau368/p-tau181 or 217 (Supplementary Table 2), in individuals with symptomatic AD (Fig. 4A–D).

## Discussion

In this study, we show that tau368/t-tau is associated with AD pathophysiological and clinical features in the symptomatic phase of the disease, not entirely captured using established CSF biomarkers indicative of tau pathology. We demonstrate that by normalizing tau368 to t-tau, there is a stronger relationship with uptake of a second-generation tau PET tracer in individuals with symptomatic AD as compared to using either of the biomarkers in isolation, especially in limbic and isocortical brain regions. In contrast, in the group of CU individuals (encompassing young, CU– and CU+), no significant associations were observed between tau368/t-tau and tau PET (all *p* > 0.05), while several significant associations were seen for p-tau181, p-tau217, and t-tau, further suggesting that these biomarkers capture different aspects of tau pathology.

Further, we found that the tau368/t-tau ratio better reflects the cognitive performance, and thereby clinical disease severity in patients with symptomatic AD, as compared to p-tau181, p-tau217, and t-tau. Finally, we saw that younger individuals with symptomatic AD had higher tau PET SUVR in limbic and isocortical brain regions as well as lower tau368/t-tau.

As previously described, accumulating evidence supported both by clinical observational studies [3], as well as experimental studies on humans [26] and animals [27] suggest that increasing concentrations of CSF p-tau forms and t-tau associate with the emergence of Aβ pathology [4]. Used in conjunction with the CSF Aβ42/Aβ40 ratio, they offer excellent diagnostic accuracy and are widely used in clinical settings [28]. However, as previously mentioned, their correlation with aggregated tau, as indexed in vivo using tau PET, is confounded by their collinearity with amyloid [29]. In this study, we confirm and extend our previous finding that tau368/t-tau is altered in AD and that it correlates with tau PET in patients with symptomatic AD [15] in brain regions associated with more advanced tau pathology (corresponding to neuropathological Braak stages III–IV and V–VI), while this association was less clear for p-tau181, p-tau217, and t-tau. The associations with tau PET that are seen for CSF p-tau and t-tau in this, and many other studies [4, 5], seem to be driven by individuals early in the AD continuum, and as the disease progresses, the variability becomes greater [4]. This is also consistent with studies showing that p-tau plateaus at later stages of the disease [30]. Of note, however, tau368/t-tau was not associated with tracer SUVR in the transentorhinal brain regions, which may be explained by the fact that tau accumulation in these stages are less prominent with disease progression, as compared to brain regions affected with more advanced disease, as indicated in a previous study [20].

The non-linear relationship between tau PET tracer uptake and tau368/t-tau found in a previous study [15], and also observed here, supports that there is a variable processing of tau in healthy subjects, which is then distorted towards secreting a higher relative abundance of N- to mid-terminal tau, being later shifted to produce more C-terminal fragments (which are more prone for aggregation) as AD progresses. In addition, the correlation found with total cognition exclusively for CSF tau368/t-tau ratio may suggest that it may be reflective of clinical disease stage, rather than state, which is likely to be better captured with CSF p-tau. In agreement, tau PET has been shown to better reflect cognitive performance as compared to CSF p-tau or t-tau CSF biomarkers, as it measures tau aggregates, likely being a downstream event of soluble tau secretion [31–33]. Thus, biomarkers of soluble tau release are generally considered to poorly reflect cross-sectional degree of cognitive impairment [34]. In addition, previous studies have indicated that decreased CSF tau368/t-tau increases concordance between CSF and PET status (more cases labeled as both CSF-positive and tau PET-positive) [35] and that it may correlate more strongly with tau PET tracer uptake as compared to p-tau181 and t-tau [15]. This may be due p-tau and t-tau rather capturing the rate with which soluble tau species are secreted at a certain time-point. As an analogy, the change in Aβ homeostasis, which occurs in the AD pathogenesis, is better accounted for when the concentration of Aβ42 is normalized to Aβ40, thus accounting for interindividual differences in Aβ production and clearance [28]. In a similar manner, normalizing CSF tau368 to t-tau may lead to a better marker of tau aggregates, by correcting for shifting proteolytic processing and secretion of tau, with N- to mid-terminal tau truncated fragments being released into the CSF, and that more C-terminal species containing the aggregation-prone microtubule-binding region are retained in the core of tangles [36], and thus becoming relatively scarcer in AD. However, the concept of targeting alterations in tau processing as a fluid biomarker may not only have implications for AD, as a recent study showed that tau peptides in the microtubule-binding region (MTBR-tau_275_ and MTBR-tau_282_) when used in a ratio with a t-tau-like peptide were able to discriminate individuals with corticobasal degeneration (CBD), certain frontotemporal lobar degeneration (FTLD)-*MAPT* variants and AD, when compared with other tauopathies and clinical mimics [37]. The opportunity of more accurately stage tau pathology both in AD and other tauopathies in an affordable manner, both for clinical management and for clinical trial enrollment, as demonstrated in the donanemab phase 2 trial in AD, would be of great use. In that trial, only patients with tau pathology which could be considered as moderate as indexed by tau PET SUVR in an AD-related topographic distribution were included [38].

Further, as mentioned, younger individuals with symptomatic AD had higher tau PET load in brain regions being affected later in the disease process as well as lower tau368/t-tau, whereas this was not the case for p-tau181, p-tau217, and t-tau. Previous studies have found that neocortical tau PET tracer uptake is higher in younger individuals with AD [39, 40], further highlighting that tau368/t-tau is captures an aspect of tau pathology not well-reflected using only core CSF biomarkers. This possibly reflects the more aggressive disease course which has been observed in younger patients, as suggested by previous studies [41, 42]. As we believe that this finding was reflecting a pathophysiological feature of AD in younger individuals, we did not age-adjust our analyses.

There are limitations to this study. First, a relatively low number of participants with symptomatic AD were included, precluding firm conclusions regarding the changes of tau368/t-tau in relation to tau pathology across brain regions, as detailed in Fig. 3. Further, although [^18^F]MK-6240 is a highly sensitive in vivo marker of tau aggregates in general [9], it is also a measure of tau tangles that would best quantified using neuropathology. Also, the cross-sectional nature of this study prevents us from studying the disease progression of individual patients. A future longitudinal study would be more appropriate to model tau368/t-tau in relation to clinical disease progression (e.g., cognitive worsening and rate of tau accumulation).

To conclude, we found that tau368/T-tau captures aspects of tau pathology in the symptomatic stages of AD, not accurately reflected by the core CSF biomarkers. This could potentially influence how tau pathology is indexed using fluid biomarkers both in clinical settings, as well as in clinical trials.

## Supplementary Information

**Additional file 1: Supplementary figure 1.** Baseline CSF tau368 concentrations. **Supplementary figure 2.** Correlation between CSF tau, cognition and tau PET with age. **Supplementary figure 3.** ROC curves for identifying Braak stages. **Supplementary figure 4.** Correlation between CSF tau biomarkers and hippocampal volume. **Supplementary table 1.** Correlation between CSF tau biomarkers and tau PET SUVr. **Supplementary table 2.** Correlations with tau PET imaging and cognition using ratios with p-tau in the group with symptomatic AD. **Supplementary table 3.** The relationship between CSF p-tau217 and PET imaging. **Supplementary table 4.** The relationship between CSF p-tau181 and PET imaging. **Supplementary table 5.** The relationship between CSF t-tau and PET imaging. **Supplementary table 6.** Correlation between global cognition and CSF biomarkers in the whole group.

## Data Availability

The data from the TRIAD study will be made available from the senior authors upon reasonable request. Such arrangements are subject to standard data-sharing agreements. Of note, the data used in the present work is not publicly available because the information could compromise the participants’ privacy.
