# Supplementary material for: CSF tau368/total-tau ratio reflects cognitive performance and neocortical tau better compared to p-tau181 and p-tau217 in cognitively impaired individuals
Source: Alzheimers Res Ther. 2022 Dec 22;14:192. doi: 10.1186/s13195-022-01142-0 (PMC9773470; doi:10.1186/s13195-022-01142-0)
Supplement: Supplementary file 1 — Additional file 1: Supplementary figure 1. Baseline CSF tau368 concentrations. Supplementary figure 2. Correlation between CSF tau, cognition and tau PET with age. Supplementary figure 3. ROC curves for identifying Braak stages. Supplementary figure 4. Correlation between CSF tau biomarkers and hippocampal volume. Supplementary table 1. Correlation between CSF tau biomarkers and tau PET SUVr. Supplementary table 2. Correlations with tau PET imaging and cognition using ratios with p-tau in the group with symptomatic AD. Supplementary table 3. The relationship between CSF p-tau217 and PET imaging. Supplementary table 4. The relationship between CSF p-tau181 and PET imaging. Supplementary table 5. The relationship between CSF t-tau and PET imaging. Supplementary table 6. Correlation between global cognition and CSF biomarkers in the whole group. [file 13195_2022_1142_MOESM1_ESM.docx]

**Supplementary Material**

Simrén J. *et al.* CSF tau368/total-tau ratio better reflects cognitive performance and neocortical tau compared to p-tau181 and p-tau217 in cognitively impaired individuals

**Supplementary figure 1.** Baseline CSF tau368 concentrations.

**Supplementary figure 2.** Correlation between CSF tau, cognition and tau PET with age.

**Supplementary figure 3.** ROC curves for identifying Braak stages.

**Supplementary figure 4.** Correlation between CSF tau biomarkers and hippocampal volume.

**Supplementary table 1.** Correlation between CSF tau biomarkers and tau PET SUVr

**Supplementary table 2.** Correlations with tau PET imaging and cognition using ratios with p-tau in the group with symptomatic AD.

**Supplementary table 3.** The relationship between CSF p-tau217 and PET imaging.

**Supplementary table 4.** The relationship between CSF p-tau181 and PET imaging.

**Supplementary table 5.** The relationship between CSF t-tau and PET imaging.

**Supplementary table 6.** Correlation between global cognition and CSF biomarkers in the whole group.

**Supplementary references.**

**Supplementary figure 1.** Group comparison of CSF tau368 concentrations.


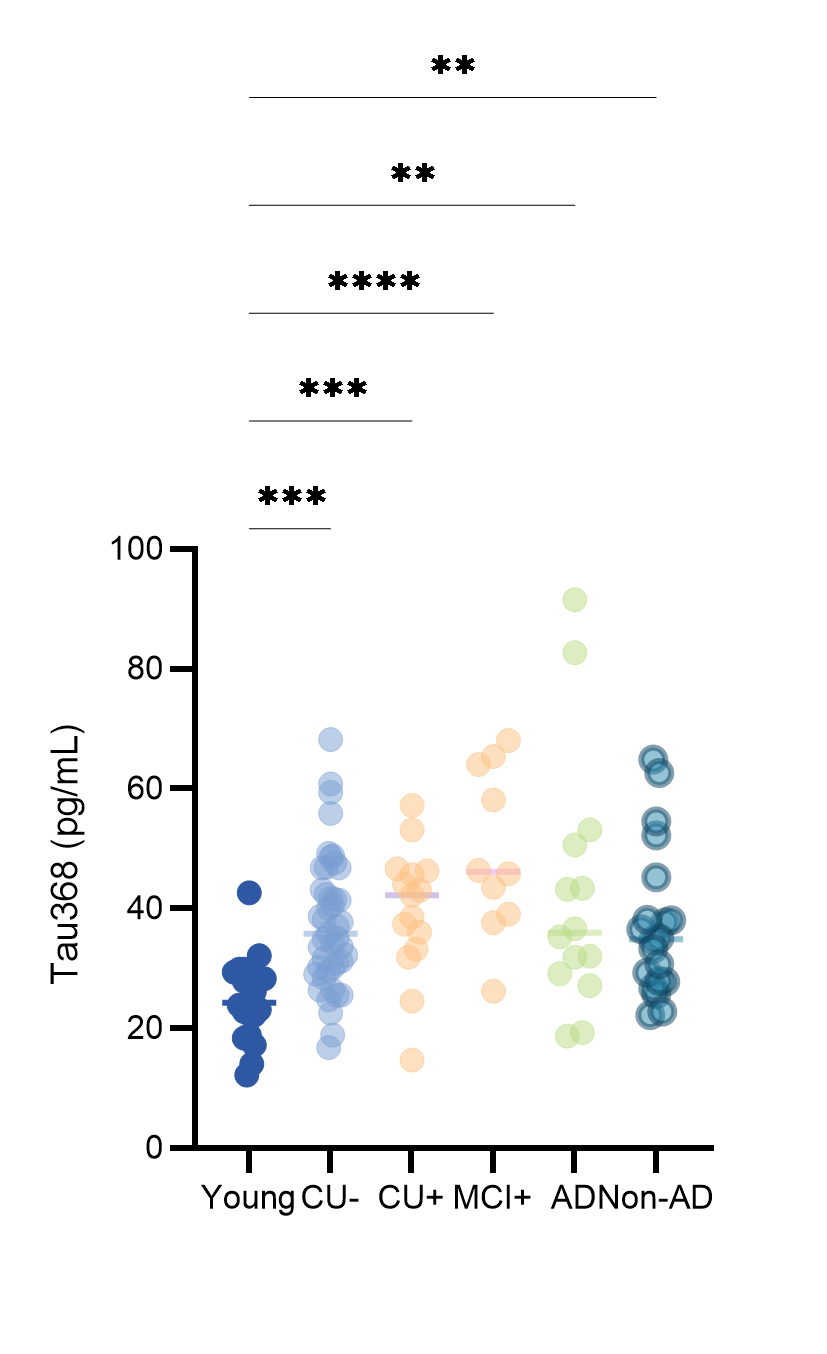


Baseline comparisons of CSF tau368 biomarker concentrations across groups. Abbreviations: AD. Alzheimer's disease; CU. cognitively unimpaired; MCI, mild cognitive impairment; CSF, cerebrospinal fluid. All *P*-values are derived from Kruskal-Wallis tests, adjusted for false discovery rate (FDR) using the Benjamini-Hochberg method.

**P*<0.05; ** *P*<0.01; *** *P*<0.001; *P*<0.0001


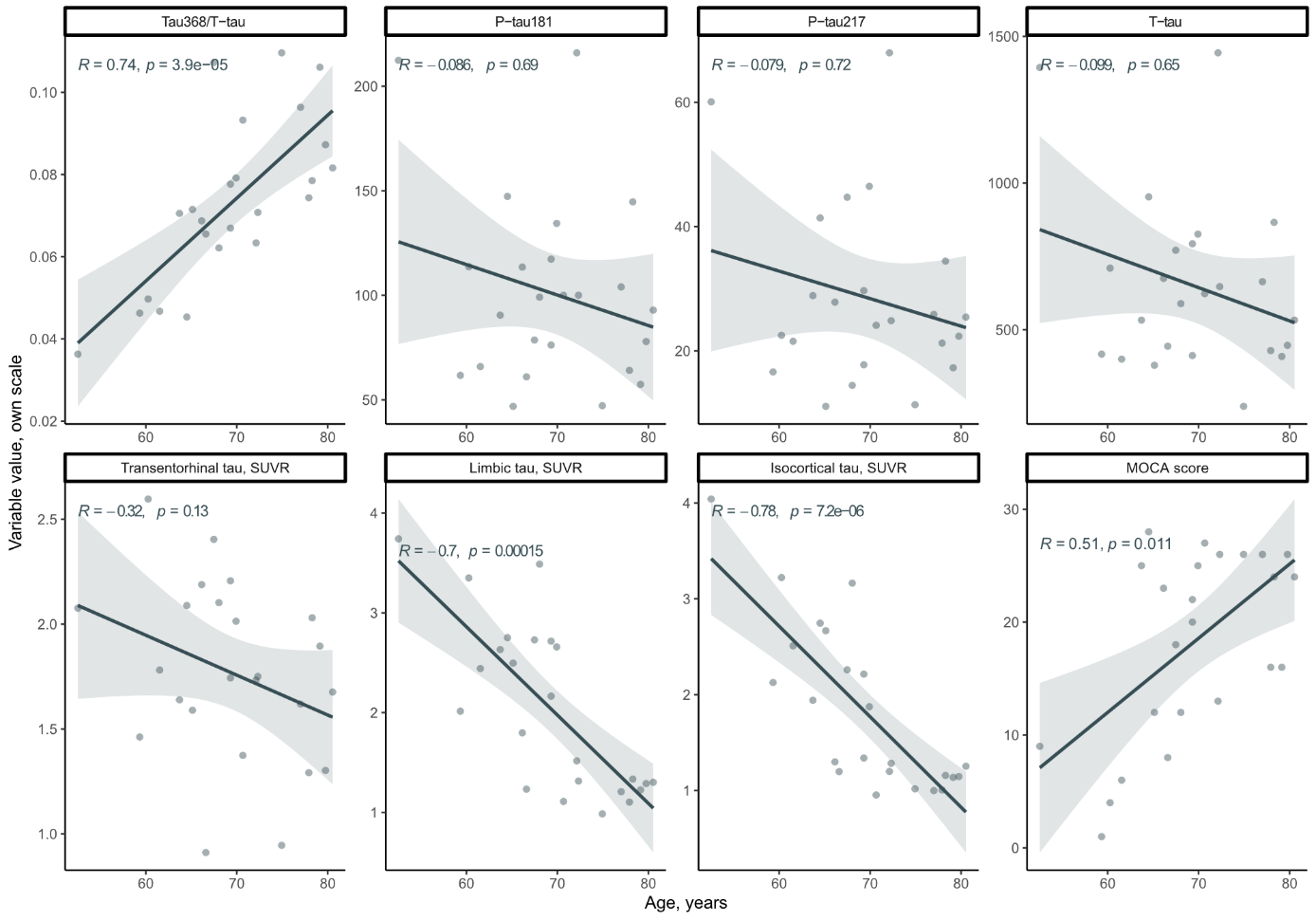
**Supplementary figure 2.** Correlation between CSF tau, cognition, and tau PET with age.

Associations between age and CSF (A) tau368/t-tau (B) p-tau217 and (C) p-tau181 and (D) t-tau in participants with symptomatic AD. Abbreviations: P-tau181/217, phosphorylated tau 181/217; T-tau, total tau; CSF, cerebrospinal fluid; MRI, magnetic resonance imaging. All *P*-values and R coefficients are derived from Spearman correlations.

**Supplementary figure 3.** Baseline CSF tau368 concentrations.


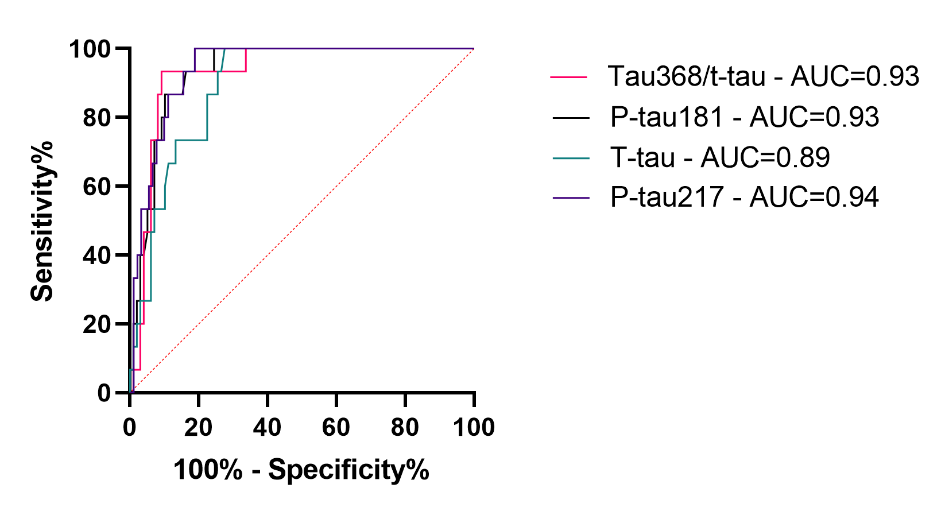

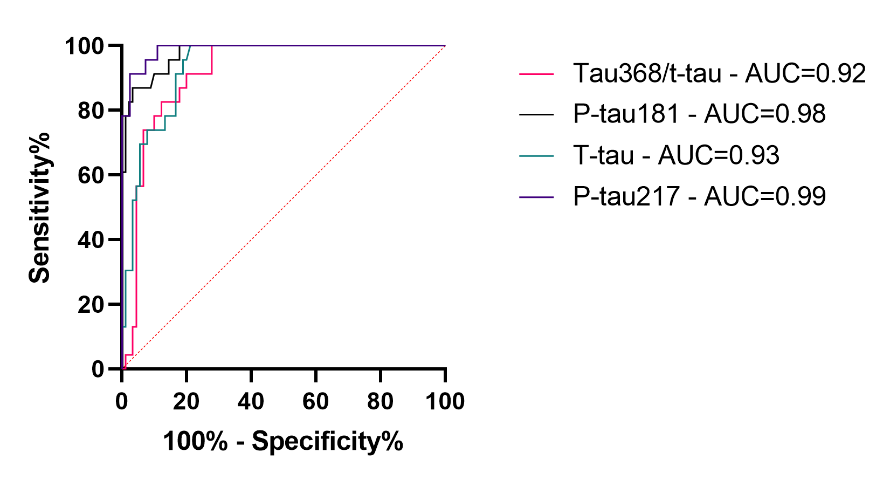

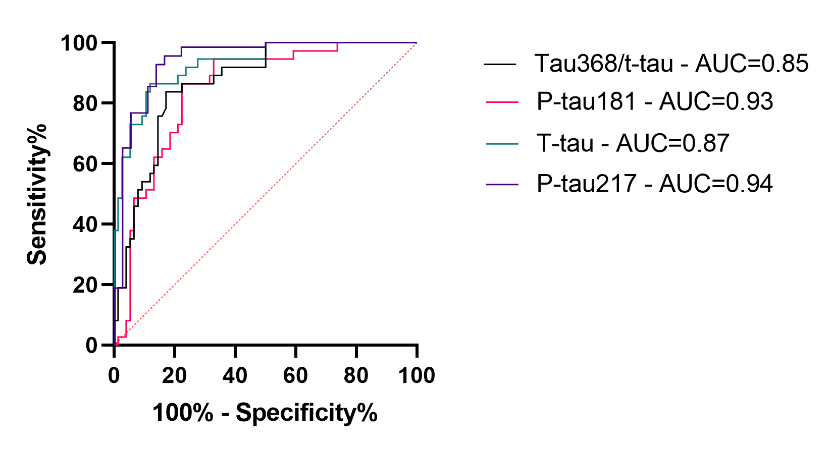


**A**

**B**

**C**

Area under the curve (AUC) for tau368/t-tau, p-tau181, t-tau and p-tau217 between Braak stages (A) I-II vs 0. (B) III-IV vs 0-II, and VI vs. 0-IV as indexed by ^18^F-MK6240 PET. Abbreviations: PET, positron emission tomography.


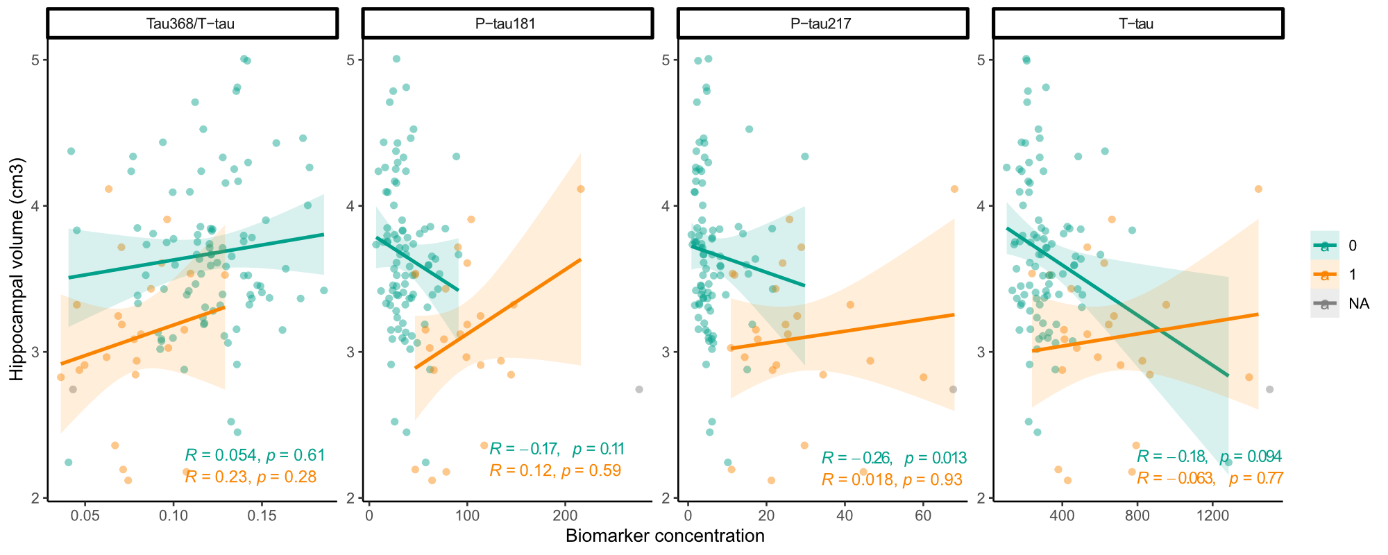
**Supplementary figure 4.** Correlation between CSF tau biomarkers and hippocampal volume.

Associations between MRI-derived hippocampal volume and CSF (A) tau368/t-tau (B) p-tau217 and (C) p-tau181 and (D) t-tau. Participants are stratified into Aβ+ MCI and AD dementia (1) vs. all others (0). Abbreviations: AD, Alzheimer's disease; MCI, mild cognitive impairment; P-tau181/217, phosphorylated tau 181/217; T-tau, total tau; CSF, cerebrospinal fluid; MRI, magnetic resonance imaging. All *P*-values and R coefficients are derived from Spearman correlations. One clear outlier, shown in grey and labeled NA, was excluded from the statistical analysis but remain in the graph.

**Supplementary table 1.** Correlation between CSF tau biomarkers and tau PET SUVr.

| CSF Biomarker | In vivo Braak stage* | Spearman Rho | P-value |
| --- | --- | --- | --- |
| Tau368/T-tau | I-II | -0.47 | < 0.0001 |
|  | III-IV | -0.47 | < 0.0001 |
|  | V-VI | -0.38 | < 0.0001 |
| P-tau181 | I-II | 0.73 | < 0.0001 |
|  | III-IV | 0.62 | < 0.0001 |
|  | V-VI | 0.44 | < 0.0001 |
| P-tau217 | I-II | 0.70 | < 0.0001 |
|  | III-IV | 0.61 | < 0.0001 |
|  | V-VI | 0.45 | < 0.0001 |
| T-tau | I-II | 0.62 | < 0.0001 |
|  | III-IV | 0.56 | < 0.0001 |
|  | V-VI | 0.38 | < 0.0001 |

*in vivo Braak stages derived from [F^18^]-MK6240 tau PET.[^1^](#_ENREF_1)

**Supplementary table 2.** Investigating other ratios in the group with symptomatic AD.

|  | Transentorhinal tau (I-II) | | Limbic tau (III-IV) | | Isocortical tau (V-VI) | | MOCA | | MMSE | |
| --- | --- | --- | --- | --- | --- | --- | --- | --- | --- | --- |
|  | **Spearman Rho** | **P-value** | **Spearman Rho** | **P-value** | **Spearman Rho** | **P-value** | **Spearman Rho** | **P-value** | **Spearman Rho** | **P-value** |
| Tau368/T-tau | -.25 | .25 | **-.58** | **.0030** | **-.67** | **<.0010** | **.53** | **.0080** | **.46** | **.025** |
| Tau368/P-tau217 | -.084 | .70 | -.35 | .10 | -.39 | .07 | .23 | .29 | .25 | .26 |
| Tau368/P-tau181 | **-.41** | **0.05** | **-.62** | **.0010** | **-.68** | **<.0010** | .37 | .080 | .28 | .19 |

**Supplementary table 3.** The relationship between CSF p-tau217 and PET imaging.

| Independent variables | | R^2^ | Adj. R^2^ | AICc |
| --- | --- | --- | --- | --- |
| Amyloid composite | | 0.07360 | 0.02949 | 128.1 |
| Transentorhinal tau (I-II) | Tau | 0.1120 | 0.06973 | 127.1 |
|  | Amyloid + Tau | 0.1514 | 0.06658 | 129.0 |
| Limbic tau (III-IV) | Tau | 0.05548 | 0.01050 | 128.5 |
|  | Amyloid + Tau | 0.1021 | 0.01230 | 130.3 |
| Isocortical tau (V-VI) | Tau | 0.02882 | -0.01743 | 129.2 |
|  | Amyloid + Tau | 0.08297 | -0.008730 | 130.8 |

R^2^ and adjusted R^2^ are derived from linear regression models. Akaike information criteria with correction for small sample sizes (AICc) was calculated to determine the best fitting model, accounting for the complexity of the model. An AIC <2 compared to another model indicates less information loss, and thus a better model.

**Supplementary table 4.** The relationship between CSF p-tau181 and PET imaging.

| Independent variables | | R^2^ | Adj. R^2^ | AICc |
| --- | --- | --- | --- | --- |
| Amyloid composite | | 0.04001 | -0.003629 | 188.0 |
| Transentorhinal tau (I-II) | Tau | 0.1675 | 0.1296 | 184.6 |
|  | Amyloid + Tau | 0.1762 | 0.09780 | 187.2 |
| Limbic tau (III-IV) | Tau | 0.09440 | 0.05324 | 186.6 |
|  | Amyloid + Tau | 0.1079 | 0.02298 | 189.1 |
| Isocortical tau (V-VI) | Tau | 0.06982 | 0.02754 | 187.2 |
|  | Amyloid + Tau | 0.08592 | -0.001135 | 189.7 |

R^2^ and adjusted R^2^ are derived from linear regression models. Akaike information criteria with correction for small sample sizes (AICc) was calculated to determine the best fitting model, accounting for the complexity of the model. An AIC <2 compared to another model indicates less information loss, and thus a better model.

**Supplementary table 5.** The relationship between CSF t-tau and PET imaging.

| Independent variables | | R^2^ | Adj. R^2^ | AICc |
| --- | --- | --- | --- | --- |
| Amyloid composite | | 0.04953 | 0.006332 | 278.3 |
| Transentorhinal tau (I-II) | Tau | 0.1852 | 0.1482 | 274.6 |
|  | Amyloid + Tau | 0.1976 | 0.1212 | 277.1 |
| Limbic tau (III-IV) | Tau | 0.1091 | 0.06856 | 276.7 |
|  | Amyloid + Tau | 0.1267 | 0.04357 | 279.2 |
| Isocortical tau (V-VI) | Tau | 0.08454 | 0.04293 | 277.4 |
|  | Amyloid + Tau | 0.1048 | 0.01950 | 279.8 |

R^2^ and adjusted R^2^ are derived from linear regression models. Akaike information criteria with correction for small sample sizes (AICc) was calculated to determine the best fitting model, accounting for the complexity of the model. An AIC <2 compared to another model indicates less information loss, and thus a better model.

**Supplementary table 6.** Correlation between global cognition and CSF biomarkers in the whole group.

| Biomarker | Spearman Rho | P-value |
| --- | --- | --- |
| Tau368/T-tau | 0.43 | <0.0001 |
| P-tau217 | -0.55 | <0.0001 |
| P-tau181 | -0.52 | <0.0001 |
| T-tau | -0.49 | <0.0001 |

*Global cognition as indexed with Montreal cognitive assessment (MoCA)

**Supplementary references**

1. Pascoal TA, Therriault J, Benedet AL, et al. 18F-MK-6240 PET for early and late detection of neurofibrillary tangles. *Brain*. Jul 16 2020;doi:10.1093/brain/awaa180
